# The pieces are already built: converging India's national assets to close the mental health treatment gap

**DOI:** 10.3389/fdgth.2026.1915080

**Published:** 2026-08-05

**Authors:** Shyamkumar Sriram

**Affiliations:** Department of Rehabilitation and Health Services, College of Public Affairs and Health Sciences, University of North Texas, Denton, TX, United States

**Keywords:** ASHA, ayushman bharat digital mission, digital mental health, India, task-sharing, tele MANAS, treatment gap, universal health coverage

## Abstract

India carries one of the world's largest mental health treatment gaps. The National Mental Health Survey found that about one in ten adults lives with a mental disorder, yet roughly five in six receive no care; the country has fewer than one psychiatrist per 100,000 people; and mental illness is projected to cost about US$1.03 trillion over two decades. No specialist-led model can close a gap of that size. This Perspective argues that India is nonetheless unusually well placed to close it, because it has already assembled, and already funds, components that most low- and middle-income countries still lack. Indian trials, including a national-scale cluster randomized study, show that lay counselors and community health workers, supported by simple digital tools, deliver effective treatment for depression in routine primary care. India has built a national digital health backbone in the Ayushman Bharat Digital Mission, a national tele-mental-health service in Tele MANAS, and a district mental health program that reaches most of the country, and it is beginning to govern artificial intelligence in health. The problem is that these assets largely run in parallel. The unfinished task is convergence: routing task-shared, digitally supported care through the existing backbone rather than launching fresh pilots. I set out a three-layer model where shared infrastructure lowers the cost of adding mental health, a task-shared workforce makes delivery affordable, and domestic financing and rights-based governance make it durable. Connectivity, literacy, and gender divides mean technology must augment human care, not replace it.

## Introduction

India has one of the widest mental health treatment gaps in the world. The National Mental Health Survey, the country's only nationwide survey, found that about 10.6 percent of adults currently live with a mental disorder and put the overall treatment gap at 84.5 percent, rising above 85 percent for common conditions such as depression and anxiety (1). The costs land in both lives and money: suicide is among the leading causes of death in young Indians, and mental illness is projected to cost the country about US$1.03 trillion between 2012 and 2030 (2). The supply side explains the gap. India has only about 0.75 psychiatrists per 100,000 people, roughly nine thousand for a population of 1.4 billion, well short of what the field considers adequate, and most of them practice in cities while the burden falls hardest on rural districts (3). The arithmetic is unforgiving: training enough specialists to meet the need is neither affordable nor fast enough for the people who need care today.

Digital technology is the usual prescription, and the evidence has matured beyond early enthusiasm. A meta-analysis of randomized trials across low- and middle-income countries found that digital mental health interventions produce moderate reductions in depressive and anxiety symptoms (4). The more important point for India is that these tools work best as a multiplier of people rather than a replacement for them. What sets India apart is that it has already built, and already funds, the components this approach requires. The task is no longer to invent a solution but to connect the assets that already exist. This Perspective sets out what that convergence would involve, why India is unusually positioned to achieve it, and what stands in the way.

### India already has the evidence

Task-shared psychological care is not a hypothesis in India; it is a tested intervention with national-scale evidence. The Healthy Activity Program, a brief behavioral treatment for depression delivered by trained lay counselors in primary care in Goa, outperformed enhanced usual care for moderate-to-severe depression and was cost-effective (5). The strongest evidence comes from the SMART Mental Health trial, which paired a clinical decision-support app with the accredited social health activists, the ASHAs who already serve every village, and a community anti-stigma campaign. In a cluster randomized trial across 44 primary health centers in Andhra Pradesh and Haryana, with 9,928 participants drawn from a screening base of roughly 170,000 adults, remission among high-risk participants reached 74.7 percent at twelve months in the intervention arm against 50.6 percent in controls (odds ratio 2.88), alongside reductions in anxiety and self-harm risk and a measurable fall in stigma (6). These are Indian trials, run inside the government health system, and they show that a digitally supported, task-shared model can deliver effective care at the front line.

### India already has the national assets

India has also built the delivery and data infrastructure that most countries still lack. Tele MANAS, the national tele-mental-health service launched in October 2022, had handled close to three million calls through 53 cells across all 36 states and union territories within about three years, operating round the clock in 20 languages, with a mobile application, a video-consultation option, and counselors supervised by specialists, financed entirely from the domestic budget (7). The District Mental Health Program already reaches more than 750 of the country's districts, embedding mental health in primary care (7). Above these sits the Ayushman Bharat Digital Mission, a domestically financed national backbone built on open standards and reusable digital public goods, which by 2026 had issued more than 90 crore (over 900 million) digital health accounts spanning all 36 states and union territories and connected hundreds of public and private providers, explicitly to support universal health coverage (8, 9). India has also begun to govern the next layer, issuing national ethical guidance for the use of artificial intelligence in healthcare rather than letting it arrive ungoverned (10). Few low- and middle-income countries can claim a national tele-mental-health service, a near-universal district mental health program, and an interoperable national health backbone at once. India has all three.

The weakness is not capability but coherence. These assets were built at different times by different bodies, and they largely run in parallel. A call to Tele MANAS does not automatically reach a person's record in the Ayushman Bharat backbone; an ASHA's screening app may not write to the same system a district psychiatrist uses; and effective task-sharing models proven in Indian trials are not yet routed through the national infrastructure that could carry them everywhere.

### The unfinished task is convergence

**Figure 1:**
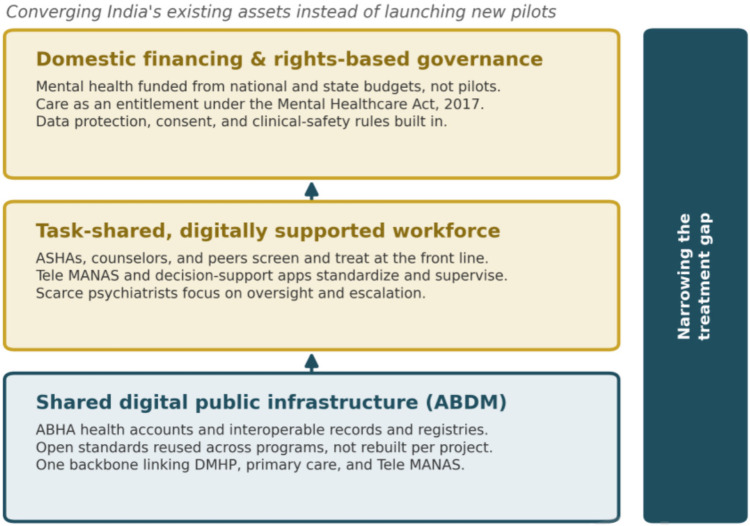
A three-layer model for converging India's existing assets. Shared digital public infrastructure (the Ayushman Bharat Digital Mission) lowers the cost of adding a mental health module; a task-shared, digitally supported workforce of ASHAs, counselors, and peers, backed by Tele MANAS and by private specialists reachable through the Health Professional Registry and a teleconsultation tariff, makes delivery affordable; and domestic financing with rights-based governance under the Mental Healthcare Act, 2017 makes it durable.

The agenda for India, summarized in Figure 1, is therefore convergence rather than invention. On a shared backbone, adding mental health means writing to existing standards rather than procuring a new vertical system; the validated screening and decision support already tested in the SMART Mental Health trial can ride on records clinicians use; Tele MANAS can serve as the escalation and specialist-support layer behind front-line workers; and a person's care can follow them across a helpline call, an ASHA visit, and a district clinic (11). Those standards are already defined. The Ayushman Bharat Digital Mission is built on HL7 FHIR Release 4 for data exchange, with SNOMED CT and ICD-10 as its clinical terminologies and LOINC for investigations, specified in the national FHIR implementation guide maintained by the National Resource Centre for EHR Standards (12). A minimal mental health dataset is therefore not a new architecture but a small set of FHIR-conformant profiles and value sets: a coded screening result such as a PHQ-9 or GAD-7 score, a SNOMED CT or ICD-coded provisional diagnosis, a risk flag, and a referral element. Coding the ASHA's screening entry and the Tele MANAS psychiatrist's diagnosis in the same terminology is what allows a score captured on a village doorstep to be read, unambiguously, by a specialist on a helpline. The marginal cost of adding mental health to infrastructure the country already runs is far lower than the cost of standing up a separate program, which is what makes durable coverage affordable. The same logic extends to perinatal mental health, where a digitally enabled, peer-delivered version of the Thinking Healthy Program matched specialist-supported care in trials and was developed through adaptation work spanning India and Pakistan (13).

**Figure 2:**
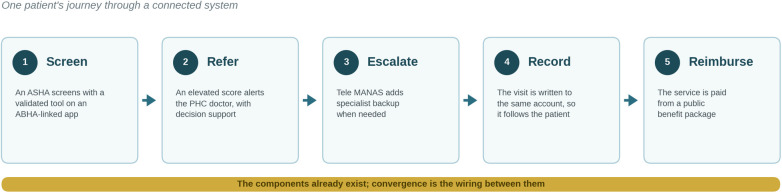
The convergence pathway: a single patient's journey through a connected system, from screening by a community health worker on an app linked to the ayushman bharat health account, through physician review and specialist backup via tele MANAS, to a shared record and reimbursement through a public benefit package. The components already exist; convergence is the wiring between them.

A concrete pathway shows what convergence looks like in practice (Figure 2). An ASHA screens an adult with a brief, validated questionnaire on an app linked to that person's health account; an elevated score flags the case to the primary care doctor and, where needed, to Tele MANAS for specialist backup; the counseling that follows is recorded against the same account, so the next provider sees it; and the service is reimbursed through a publicly funded benefit package rather than a project grant. Every component in that sequence already exists in India. What is missing is the wiring between them, a defined set of interoperable mental health data elements and referral pathways, and that wiring, rather than another pilot, is the intervention.

Convergence must also reach the private sector, where most of India's psychiatrists work and most specialist capacity sits. The public system anchors front-line delivery, but the scarce specialists needed for the escalation tier are concentrated in urban private practice, and a model that ignores them forfeits the very expertise task-sharing depends on. Two existing mechanisms can bring them in without new institutions. First, the Health Professional Registry lets private psychiatrists register verifiable credentials in the same backbone, so a district team can discover and route to them. Second, the publicly funded benefit package can reimburse specialist teleconsultation and supervision, paying private psychiatrists for the hub-and-spoke backup that Tele MANAS and district services need rather than relying on their goodwill (14). Empanelment and a teleconsultation tariff turn a fragmented private specialist base into reachable escalation capacity.

### Finance and govern it as a public good

Sustainability depends on treating digital mental health as a recurring commitment rather than a project. India is already moving in that direction: the national tele-mental-health program is funded from the domestic budget, and recent allocations have raised the priority given to mental health within the health envelope (7). The financing vehicle must be the right instrument, however. India's flagship insurance scheme, Pradhan Mantri Jan Arogya Yojana, reimburses secondary and tertiary hospitalisation to empanelled facilities, not the outpatient, community-based care that task-sharing delivers; comprehensive primary care, including mental health, runs instead through the Health and Wellness Centres (14). Durable financing therefore needs two distinct lines rather than one. The first is an outpatient service tariff that pays for task-shared psychological care as a defined primary-care entitlement, so delivery does not depend on time-limited grants. The second is a dedicated frontline incentive: ASHAs are honorary workers who already carry some thirty activities and more than forty task-based incentives across national programmes, and adding mental health without a matching, predictable incentive would either go uncompensated or crowd out existing work (15). Reimbursing a facility is not the same as compensating the worker who does the screening, and convergence has to fund both.

India also has a foundation many countries lack, since the Mental Healthcare Act of 2017 frames access to care as a legal entitlement (16). That foundation carries obligations, and embedding mental health data in shared infrastructure raises real and specific risks. A record of psychiatric care is stigmatising in ways a record of hypertension is not, and mental illness in India can translate into discrimination in employment, insurance, and marriage, so a health account that made such a history portable could, if poorly governed, become an instrument of exclusion rather than care (17). Three features of India's system are designed to prevent that, and each must be enforced rather than assumed. The Ayushman Bharat Digital Mission is federated and consent-first: records stay with the originating facility and move only on explicit, time-bound, revocable consent through a consent manager, with no central store to breach or repurpose (8). The Mental Healthcare Act gives statutory force to a right to confidentiality and a right to equality and non-discrimination for people with mental illness. And the Digital Personal Data Protection Act, 2023 governs how personal data may be processed, with obligations of purpose limitation, consent, and breach notification (18). The gap to watch is that this last Act does not create a special protected category for health or mental health data, so purpose limitation, strict access controls, and audit logging for mental health elements have to be built into the data standard itself, alongside clear accountability when an app or algorithm informs a clinical decision (11).

## Discussion

The case is strong but conditional, and the condition is equity. India's digital divide runs along the same lines as its treatment gap: connectivity, affordability, digital literacy, and access for women are all lower in the rural areas where unmet need is greatest (19), and the survey itself found a higher burden and lower access in rural than urban India (1). These divides are not uniform across the country. Digital readiness ranges from states such as Kerala, with roughly three-quarters digital literacy, to poorer northern states where far fewer rural women have ever used the internet; the National Family Health Survey records internet use among women below a quarter in several states against a majority in the most connected ones (20). Fiscal capacity varies just as sharply: per-capita state government health spending differs several-fold, from the lowest-spending states to those spending around four times as much (21), and ASHA density and honoraria are set state by state (15). A model that assumes a single national readiness would therefore widen inter-state inequity even as it narrowed the national gap. Convergence has to be sequenced to state capacity, beginning where digital and fiscal readiness are highest, with central support, the shared backbone, Tele MANAS, and incentive top-ups, deployed deliberately to keep lower-capacity states from falling further behind. This is also why the model keeps a human worker, an ASHA, a counselor, or a peer, as the point of contact, with technology behind them rather than in front of the patient, so that care stays reachable for people who are offline, have limited literacy, or are wary of devices.

Language and culture set a further condition. Tele MANAS operates in about twenty languages, but India has twenty-two scheduled languages and many more dialects, and screening tools and counselling scripts carry idioms of distress that do not translate directly; each must be linguistically and culturally validated before local rollout, as the Healthy Activity Program, SMART Mental Health, and Thinking Healthy adaptations show is feasible but not automatic (5, 6, 13). This is a further reason the point of contact stays a local worker who shares the patient's language and context.

Two caveats temper the argument. First, the trial evidence for digitally supported task-sharing in India, while genuinely strong, still comes from a limited number of districts, and effectiveness at national scale is not guaranteed by results in a research setting; adding a task to an overstretched worker can also dilute quality unless it displaces lower-value work or is matched by support, a risk the SMART Mental Health trial contained by giving ASHAs a simplified app that structured rather than added to their task (6). Convergence must therefore be paired with implementation research, not assumed. Second, integrating systems is a political and organizational task as much as a technical one; experience with India's own front-line digital tools shows that durability depends on data quality, workflow fit, and the human relationships that keep a tool alive in routine use, not on the software alone (22).

Three priorities follow for the near term. The first is to define and adopt a minimal set of interoperable mental health data elements, expressed as FHIR profiles and value sets coded in SNOMED CT and ICD, so that screening, referral, and follow-up can move cleanly across the Ayushman Bharat backbone, Tele MANAS, and district services. The second is to fund task-shared psychological care through two durable public lines, an outpatient primary-care tariff and a dedicated frontline incentive, so that both the service and the worker who delivers it have a recurrent and predictable source of funds. The third is to embed independent implementation and economic evaluation in every phase of scale-up, sequenced to state capacity, so that national rollout is guided by evidence rather than assumption.

There is a wider lesson. As external development assistance for health contracts sharply, many low- and middle-income countries can no longer sustain mental health through donor-funded pilots (23). India's path is instructive precisely because it is domestically financed and infrastructure-first: a country can build the backbone, the workforce model, and the legal basis itself, and then converge them. For India, the unfinished task is not to prove that digital, task-shared mental health works, which its own trials have done and which aligns with the World Health Organization's task-sharing guidance (5, 6, 24). It is to route that proven model through the national assets it has already built, and to fund and govern it as a permanent part of the country's commitment to universal health coverage.

## Data Availability

The original contributions presented in the study are included in the article/Supplementary Material, further inquiries can be directed to the corresponding author.
